# Influence of Heat Treatment and Lactic Acid Fermentation on the Physical and Chemical Properties of Pumpkin Juice

**DOI:** 10.3390/molecules29194519

**Published:** 2024-09-24

**Authors:** Emilia Janiszewska-Turak, Katarzyna Rybak, Dorota Witrowa-Rajchert, Katarzyna Pobiega, Anna Wierzbicka, Szymon Ossowski, Joanna Sękul, Aniela Kufel, Aneta Wiśniewska, Urszula Trych, Justyna Szczepańska-Stolarczyk, Andrzej Krzykowski, Anna Gramza-Michałowska

**Affiliations:** 1 Department of Food Engineering and Process Management, Institute of Food Sciences, Warsaw University of Life Sciences—SGGW, 159C Nowoursynowska St., 02-787 Warsaw, Poland; katarzyna_rybak@sggw.edu.pl (K.R.); dorota_witrowa_rajchert@sggw.edu.pl (D.W.-R.); annawierzbicka19@wp.pl (A.W.); s206700@sggw.edu.pl (S.O.); s212971@sggw.edu.pl (A.K.); s212994@sggw.edu.pl (A.W.); 2 Department of Food Biotechnology and Microbiology, Institute of Food Sciences, Warsaw University of Life Sciences—SGGW, 159C Nowoursynowska St., 02-787 Warsaw, Poland; katarzyna_pobiega@sggw.edu.pl (K.P.); s212132@sggw.edu.pl (J.S.); 3 Department of Fruit and Vegetable Product Technology, Institute of Agricultural and Food Biotechnology—State Research Institute, 36 Rakowiecka Street, 02-532 Warsaw, Poland; urszula.trych@ibprs.pl (U.T.); justyna.szczepanska@ibprs.pl (J.S.-S.); 4 Department of Thermal Technology and Food Process Engineering, University of Life Sciences in Lublin, 31 Głęboka St., 20-612 Lublin, Poland; andrzej.krzykowski@up.lublin.pl; 5 Department of Gastronomy Science and Functional Foods, Faculty of Food Science and Nutrition, Poznan University of Life Sciences, Wojska Polskiego 31, 60-624 Poznan, Poland

**Keywords:** pumpkin, carotenoids, lactic acid fermentation, heat treatment, antioxidant properties

## Abstract

Pumpkin is a highly nutritious plant, rich in valuable nutrients that benefit human health. Due to the high perishability of this fruit, the production of pumpkin juice is a practical way to use it effectively. Recently, fermented vegetable juices have been used as a dairy alternative due to their nutritional and potential probiotic properties. This study investigated the fermentation of pumpkin juice using different strains of lactic acid bacteria (LAB), with and without heat treatment. The effects of fermentation on microbial growth, pH, acidity, extract, sugars, carotenoids, polyphenols, and antioxidant properties were analyzed. The heat-treatment process did not greatly impact the dry matter content, pH, acidity, extract, or sugar content. However, it led to a reduction in carotenoid and polyphenol levels. During fermentation, there was a consistent decrease in pH and an increase in total acidity, with no noticeable differences between bacterial strains regarding their influence on these parameters. The study revealed that there were no distinctions between LAB strains in their effects on pH, acidity, and carotenoid content in fermented pumpkin juice. Nonetheless, both *L*. *sakei* and *L*. *plantarum* proved to be effective in the fermentation process, with *L. sakei* demonstrating greater adaptability. The expected pH, acidity, and sugar content changes were consistently observed throughout the fermentation process. Overall, results confirm the efficacy of the used *Lactobacillus* strains in fermenting pumpkin juice and highlight the potential impact of heat treatment on the nutritional composition of the juice. The purpose of thermal processing of pumpkin juice, which is conducted with lactic acid fermentation, is crucial for the food industry. It extends the product’s shelf life, improves its nutritional and taste profiles, and guarantees its microbiological safety.

## 1. Introduction

Pumpkins are a type of winter squash rich in vitamins and minerals, including high levels of beta-carotene, vitamin C, and vitamin E, which are essential for maintaining good vision and are powerful antioxidants, aiding in the fight against free radicals in the body [1,2,3]. However, pumpkin fruits are difficult to eat whole due to their tendency to spoil quickly. That is the reason for using pumpkins in another form than whole vegetables. Hence, pumpkin juice is a beneficial use of the fruit as it offers potential health benefits, including antioxidant, antidiabetic, and antihypertensive properties [4]. Furthermore, when combined with mango, orange, strawberry, and green apple fruits, pumpkin juice can provide essential nutrients like beta-carotene, vitamins, minerals, and antioxidants, making it suitable for functional beverages with enhanced health-promoting qualities [5]. A study conducted by Sun et al. [6] proved that pumpkins can be an excellent matrix for the growth of lactic acid bacteria. This knowledge provides the opportunity to create new products from pumpkin, improving the degree of utilization and, at the same time, giving new nutritional qualities.

The popularity of fermented fruit and vegetable juices has been noted for several years due to growing health awareness and changing lifestyle trends. Their popularity in the scientific world is also linked to their nutritional value and potential for use as an alternative to dairy products [7,8]. Consumers who limit their intake of products containing lactose, cholesterol, or certain colorings may find substitutes for many products, precisely in fermented vegetable juices. With the search for alternative sources of probiotics in food, more and more research is being conducted on using different strains of lactic acid bacteria. Research in this area could lead to probiotic products with different sensory properties [6,9,10,11].

In the food industry, it is crucial to ensure that food production processes are executed in a way that allows for consistent repetition of steps and careful control of variables. This is essential to achieve reproducible results with a high level of accuracy. The lactic acid fermentation process can be controlled by adding specific strains of lactic acid bacteria (LAB) [6]. Fermenting vegetables with selected lactic acid bacteria (LAB) can produce high-quality products with enhanced nutritional and sensory attributes. For pumpkin juices, different types of LAB were used. The study provided by Sun et al. [6] investigated the potential of fermenting pumpkin juice using various lactic acid bacteria strains, including *Lacticaseibacillus casei* (Lc), *Lactiplantibacillus plantarum* (Lp), *Lactobacillus acidophilus* (La), *Lactobacillus helveticus* (Lh), and *Lacticaseibacillus paracasei* (Lpc). Their findings suggested that La and Lh might be particularly well-suited for producing a functional beverage with an appealing flavor. In another study, *Lactobacillus mali* K8, obtained from water kefir grains showed promise as a probiotic candidate for fermenting pumpkin fruit puree [12]. Additionally, a study developed fermented pumpkin pulp using *Lactobacillus casei* KN 291, resulting in a product with high sensory acceptance and increased bacterial count, particularly with the addition of inulin [13]. Another type of lactobacillus strain (*L*. *helveticus*) has also been mentioned as a good LAB to ferment carrot, broccoli, kale, and pumpkin juices [14]. Also, *Lactobacillus fermentum* NCDC 141 was used for pumpkin juice fermentation [15]. In the literature, *L. plantarum* and *L. brevis* are the most commonly used species for fermenting pumpkin juices and other vegetable juices [1,16,17,18]. Both are heterofermentative and can produce lactic acid, ethanol, and carbon dioxide during fermentation. *L. plantarum* is known for its robustness and versatility compared to *L. brevis*. Moreover, applying *Lactobacillus plantarum* as a starter culture strain for vegetable fermentation can result in acidification and, as a result, vegetable juice fermentation. According to the literature, the following bacterial species can be used for the fermentation of pumpkin juice: *Latilactobacillus sakei*, which is commonly found in fermented foods such as kimchi; *Limosilactobacillus reuteri*, which is capable of producing an antimicrobial substance called reuterin; and *Fructilactobacillus fructivorans*, which is renowned for its capacity to ferment fructose [19,20,21]. *L. sakei* can grow in environments with high salt concentrations and low temperatures, outcompeting other bacteria and improving the texture of fermented products. Furthermore, it is believed to be beneficial in treating obesity, inflammatory bowel disease, and atopic dermatitis [22,23,24]. *L. reuteri* has been demonstrated to possess probiotic characteristics, including the ability to alleviate infantile diarrhea, reduce cholesterol levels, and defend against *Helicobacter pylori* infection. This species has been classified as safe by the European Food Safety Authority (EFSA) [25,26,27]. Fructophilic LAB (FLAB) represents a distinct group of LAB, to which *F*. *fructivorans* belongs. This species can produce antimicrobial compounds employed for food preservation and use mostly fructose as a carbon source [28,29]. Due to the properties of this LAB and the potential to ferment sugars from food-grade pumpkin juice, two of the most commonly used strains were selected for pumpkin juice fermentation, as well as those mentioned above, not previously used.

In the food industry, the heat treatment process reduces the microbial load in food and beverages, ensuring their safety and extending shelf life without significantly altering their nutritional and sensory properties. Regarding vegetable juices, the heating process generally involves heating the juice to a temperature ranging from 70 °C to 90 °C for a specific duration. The primary goal is to eradicate pathogenic microorganisms that cause foodborne illnesses, thereby significantly extending the shelf life of the juices by preventing spoilage caused by microorganisms [30,31]. The industry standard requires thermal processing for low-acid products with a pH greater than 4.6, such as pumpkin juice. Pumpkin juice is a valuable source of vitamins A and C, antioxidants, and other nutrients, most of which are preserved through pasteurization. Furthermore, heat treatment deactivates certain enzymes that could lead to spoilage and diminish the quality of pumpkin juice [31,32].

This study aimed to assess the influence of a specific *Lactobacillus* strain on the lactic acid fermentation process of pumpkin juice. In order to achieve this objective, strains with the capacity to ferment vegetable products were selected with great care and parameters such as the number of lactobacilli, pH, acidity, and sugar content were monitored. The extract was analyzed to ascertain that the sugar content underwent a reduction during the fermentation process. Furthermore, the type of sugar present in the raw material was also investigated. Also, key attributes that impact consumers’ purchasing decisions were examined, including color, antioxidant capacity, and total carotenoids. The initial research hypothesis proposed that each selected strain would yield comparable fermentation outcomes when employed for the fermentation of pumpkin juice.

According to the food industry requirements for low-acidic juices, such as heat treatment prior to processes, half of the samples were also subjected to heat treatment to deactivate juice enzymes and the indigenous microbiota. The second objective of the research was to gain insight into the impact of heat treatment on the physical and chemical parameters analyzed, as well as the number of lactic acid bacteria present. The second hypothesis was that the heat treatment process would have no significant effect on the physical and chemical parameters of the fermented pumpkin juice.

## 2. Results

### 2.1. Microbiology

**Figure 1:**
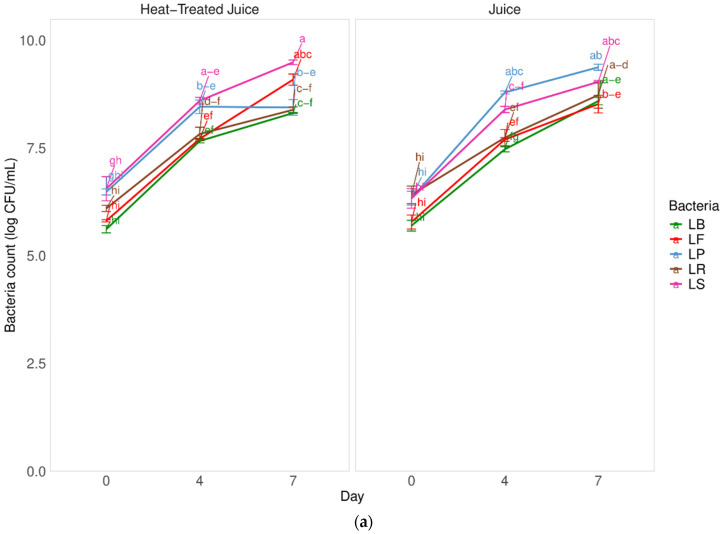

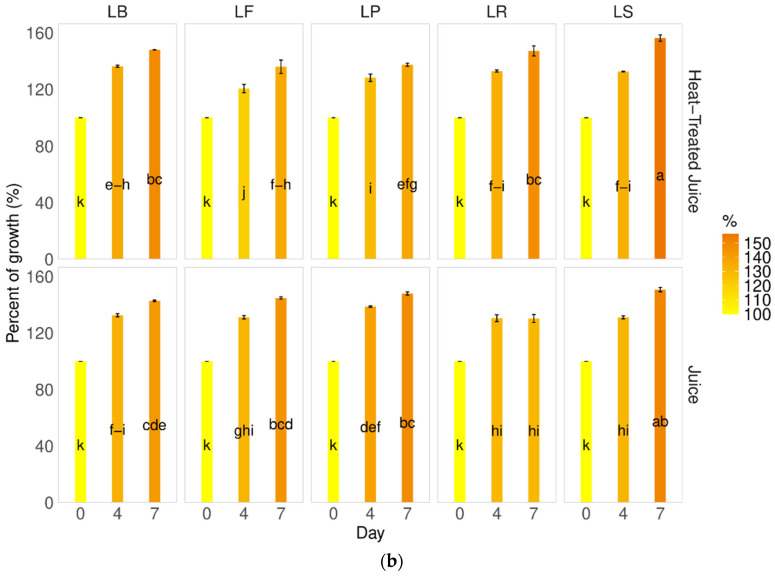
Influence of heat treatment and fermentation process on the (**a**) bacteria count in juices; (**b**) percent of lactobacillus bacteria growth in comparison to the start day. LS—*Latilactobacillus sakei* ATCC 15521; LP—*Lactipalntibacillus plantarum* ATCC 4080; LB—*Levilactobacillus brevis* DSM 20053; LR—*Limosilactobacillus reuteri* DSM 17938; LF—*Fructilactobacillus fructivorans* DSM 20203; a–k—results marked with the same letters are not statistically significantly different at α = 0.05.

Fermentation is a complex process that is influenced by the type of microorganisms, the time and temperature of the process, and the type of fermented raw material. Selecting the appropriate microorganism for fermenting pumpkin, a raw material known for its high sugar content, can reduce fermentation time and overall costs. In Figure 1a, there is a comparison of the fermentation kinetics of pumpkin juice by 5 different types of lactic acid bacteria. The study also examined the impact of pasteurizing pumpkin juice on fermentation.

There were no significant differences (*p* = 0.33 with α = 0.05) in the fermentation kinetics of juices with and without heat treatment. This suggests that the low content of indigenous microbiota (at the level of 3.1 ± 0.1 log CFU/mL) in untreated juices did not negatively impact the fermentation process after inoculation with selected LAB. Furthermore, the heat treatment process did not seem to affect the components available for the bacterial pattern in juices. After heat treatment, the total number of microorganisms was below the detection level (<1.0 log CFU/mL). Among the tested strains, the *Latilactobacillus sakei* strain showed better adaptive properties and resulted in a product containing a higher number of bacteria after fermentation. Specifically, the number of bacteria increased by 3.3 log cycles CFU/mL without heat-treated juice and 2.7 log cycles CFU/mL in heat-treated juice after 7 days of fermentation. However, the fermentation process took longer compared to other studies. Figure 1b shows the increase in the number of lactic acid bacteria during fermentation. An approximately 25% increase in the number of LAB was observed after 4 days of fermentation and about a 40% increase after 7 days. These increases in live cells are not satisfactory, indicating the need to optimize the fermentation process, such as by adding more bacterial inoculum or adjusting the fermentation temperature. Previous studies found that pumpkin juices undergo a significantly faster fermentation process when exposed to *Lactobacillus casei*. After 24 h, there was an observed increase in the microorganism count of approximately 4 log CFU/mL [1].

Other studies compared the fermentation of pumpkin juices using strains of 5 species of lactic acid bacteria (LAB): *Lacticaseibacillus casei*, *Lactiplantibacillus plantarum*, *Lactobacillus acidophilus*, *Lactobacillus helveticus*, and *Lacticaseibacillus paracasei* [6]. These studies showed an even shorter fermentation time of 12 h. The highest number of lactic acid bacteria occurred when pumpkin juice was fermented using the *Lacticaseibacillus paracasei* strain, indicating its potential to efficiently utilize sugar and produce acid in the pumpkin matrix. However, all tested LAB strains were deemed suitable for the fermentation process. Studies also highlighted the impact of inoculum quantity, raw material concentration, and process temperature on the quality of fermented pumpkin puree. Optimal conditions were determined using *Lactobacillus mali*, with a 40% concentration of pumpkin puree, 8 log CFU/mL of inoculum, and a temperature of 35 °C [33]. The durability of fermented pumpkin juices was not examined in this study, but other research suggested that the microorganism number remained acceptable for nearly two weeks. Moreover, the survival rate of LAB in fermented pumpkin puree was 88% after 4 weeks of storage, underscoring the potential for stable probiotic products and the need for further research on strain selection and process optimization.

### 2.2. Selected Properties of Pumpkin Juices

#### 2.2.1. Dry Matter and Extract

The heat treatment of raw pumpkin juice had no significant effect on the dry matter and extract (Table 1). However, after several days of fermentation, there was a noticeable reduction in the extract regardless of the strain and juice treatment.

The reduction was higher in juice without heat treatment, with a decrease in extract values ranging from 2.2% (J_LB_D4) to 18.5% (J_LR_D4) after four days compared to fresh juice. For LF, LP, and LS strains, the reduction was about 12%. After seven days of fermentation, the lowest decrease (12%) was observed for pumpkin juice of strain LS; for others, the reduction of solid content was about 23–29%. Heat-treated juices showed a smaller decrease in extract values. For LF after the 4th day, the lowest decrease in the extract was observed at 7.5 (HT_J_LF_D4), while the highest after the 7th day was 14% (J_P_LF_D7). The decrease was an average of 8–10% for the other juices, independent of the day and strain.

Similarly, the reduction in dry matter values was less evident in heat-treated juices than in those without thermal treatment. After four days, the decrease in dry matter ranged from 2% (HT_J_LB_D4) to 9.14% (HT_J_LS_D4) for heat-treated juices and 11% (J_LB_D4) to 23% (J_LR_D4) for non-heat-treated juices. After seven days, the decrease was 3.5% (HT_J_LB_D7) to 13% (HT_J_LF_D7) for heat-treated juices and 10% (J_LS_D7) to 32% (J_LP_D7 and J_LR_D7) for non-heat treated juices.

The dry matter content and solid substance (extract) decreased during fermentation regardless of the strain used and thermal treatment. This decrease is mainly attributed to the growth of microorganisms. During fermentation, lactobacillus consumes sugars to produce lactic acid, reducing dry matter content [34]. Additionally, different harvest times and pumpkin varieties result in varied levels of dry matter and solid soluble substance content. Unfortunately, this variability is common in food due to factors such as plant variety, solid components, and weather. Zhang et al. [35] have tested 3 pumpkin varieties; for the same pumpkin juice variety, *Cucurbita moschata*; they obtained extract at the level of 4.5°Brix, which is lower than in our studies. They also observed the content of sugars such as sucrose (2.24 g/100 g fw), glucose (1.08 g/100 g fw), and fructose (1.72 g/100 g fw). Such high levels of fructose can result in a higher decrease during lactic acid fermentation with the LF strain, which was confirmed in our extract decrease after 7 days of fermentation.

#### 2.2.2. pH and Total Acidity

During fermentation, lactobacillus strains produce lactic acid, which increases acidity and decreases pH.

The pH decreased by about 39–42% regardless of the day, strain, or thermal treatment. Thermal treatment did not significantly change the pH values. After four days of fermentation, the pH dropped from 6.2 in fresh samples to 3.8 for untreated juices and 3.9 for heat-treated ones. After seven days of fermentation, the pH dropped to 3.65 and 3.38, respectively, for untreated and heat-treated pumpkin juices.

The pH value of pumpkin juices was 6.23–6.21, which is higher than that of fresh pumpkin juices measured by Zhang et al. [35] and lower than those presented by Sun et al. [6]. In the literature, it could be found that differences in pH values depend on the variety of pumpkin, the juice processing, and other factors.

The pH of juices decreases faster compared to lactic acid fermentation in vegetables due to the higher availability of reduced sugars [35]. Unlike in vegetable fermentation, there is no need for an osmotic process to occur in juices because all the necessary ingredients are already present in the liquid medium.

Moreover, on the fourth day, the pH stayed under 4, guaranteeing the stability and safety of the products and allowing for storage. A pH lower than 4 also restricts harmful microorganisms’ proliferation, as Shaik and Chakraborty [36] mentioned.

The various strains showed a marked difference in total acidity values, but their behavior in both juices with and without heat treatment was consistent. Samples from the fourth and seventh day of fermentation for strains LB and LR exhibited the lowest increase in total acidity, regardless of heat treatment. Strains LP and LS, on the other hand, consistently showed the highest increase in total acidity, irrespective of the day and heat-treatment (Table 1). The total acidity range for pumpkin juice without heat treatment on the fourth day was 3.46–3.73 g of lactic acid/100 g, while for heat-treated juice, it was 3.60–3.92 g of lactic acid/100 g. By the seventh day, the values had increased to 4.80–5.95 and 5.70–6.10 g of lactic acid/100 g, respectively, for juice without and with heat treatment.

An increase in total acidity during lactic acid fermentation with dedicated strains is a typical behavior in vegetable juices. Our team [8,37] observed similar tendencies in beetroot juice fermented with strains of *Levilactobacillus brevis*, *Limosilactobacillus fermentum*, and *Lactiplantibacillus plantarum*. An increase from 0.35 to 0.7–1.27 g lactic acid/100 g juice was observed [9], while for the next season, the starting total acidity in the juice was 3.76 for beetroot juice and 1.8 for carrot juice. After seven days of fermentation, total acidity increased to about 8 g/100 g juice for LB and 11.6 for LP in beetroot and carrot juices [37].

Zhang et al. [35] tested three pumpkin juice types. They observed pH in the range of 5.92 for *C. pepo*, 6.06 for *C. maschata*, and 6.83 for *C. maxima*, while the total acidity was approximately 0.27–0.33 depending on the type of pumpkin used (*C. moschata*–*C. pepo*) [35]. Similar basic values were also observed by Sun et al. [6], who fermented different pumpkin juices with different LAB strains; in their research, the pH started from 6.4 and total acidity from 0.25 g/L. After fermentation, they observed a decrease in pH to 3.7–3.2 and an increase in acidity to about 3–5 g/L. The highest increase in acidity and decrease in pH values were observed for *Lacticaseibacillus paracasei*, while the reverse situation was seen for *Lactobacillus helveticus*.

#### 2.2.3. Color

It is crucial to keep in mind that color plays a significant role in consumer preferences as we often make purchasing decisions based on visual appeal. That is the reason why we need to focus on new product design. For example, pumpkin juice, which is distinctly dark brownish orange in color, can be described by color coefficients from the Hunter LAB scale. Lightness determines the perception of the color’s depth, while the a* value represents the redness or greenness, with pumpkin juices having slightly positive values (+a*) compared to negative green colors (−a*). The b* value indicates the yellowness or blueness, with pumpkin juice orientated more towards yellow due to its carotenoid content. When modifying the juice without altering its color too much, it is essential to consider the total color difference (dE). This provides insight into potential changes compared to the original product, which can be observed by an untrained consumer [31,35,38].

After heat treatment, an increase in brightness, redness, and yellowness was observed. After fermentation, a decrease in lightness was observed for all samples, while the situation depended on the pretreatment of the juice for redness and yellowness. For heat-treated samples after fermentation, a decrease in a* and b* values was observed, while for samples without heat treatment, no obvious trends were observed (Table 2). The changes were more pronounced in heat-treated juice compared to those without heat treatment. These variations can be attributed to environmental factors, particularly the carotenoid content. In pumpkin juice literature, a higher percentage of α- and β-carotene, β-cryptoxanthin, and zeaxanthin were found [26], contributing to the yellow/orange color. Thus, the b* value is more associated with these carotenoids. Lycopene, responsible for the red color, was also identified in pumpkin juice [35], which correlates more with the a* coefficient representing the redness of the samples. Carotenoids are sensitive to acidic conditions, therefore their degradation during lactic acid fermentation explains the observed decrease in value [6,39]. An increase in fermentation time for selected strains and a decrease in redness and yellowness values were observed for juices after heat treatment, while no particular trends were observed for those without HT (Table 2).

**Figure 2:**
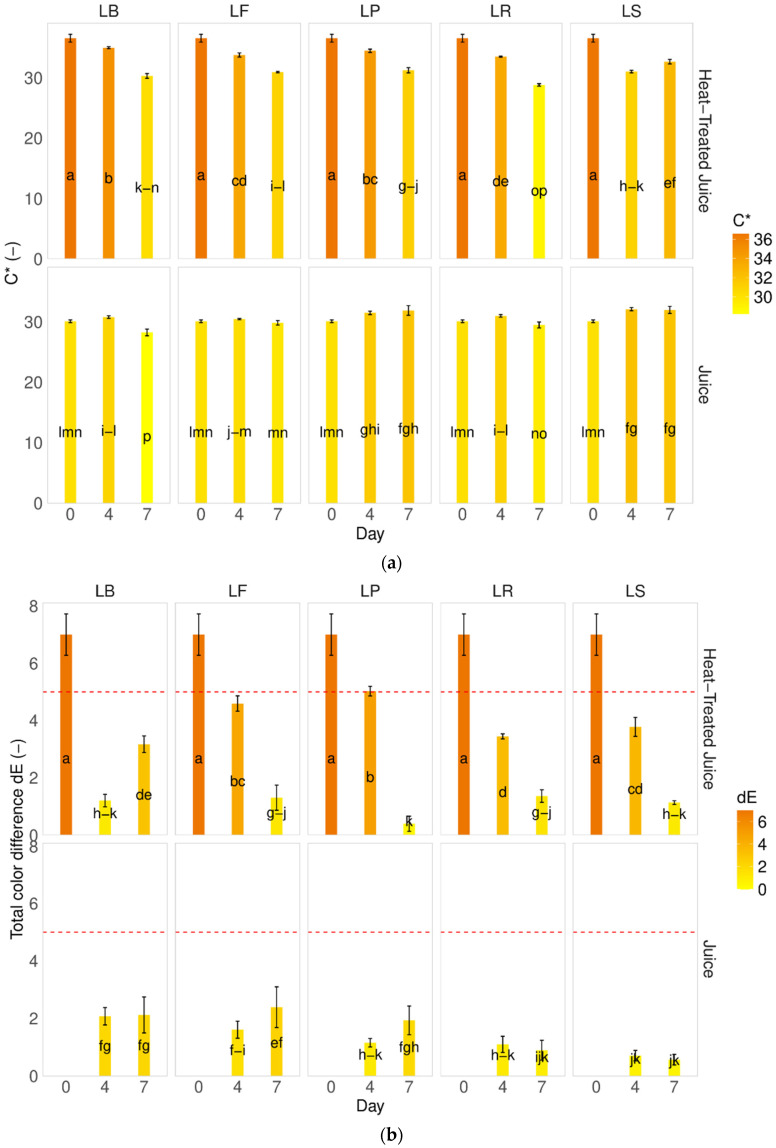
Influence of the heat treatment and fermentation on the color determinants (**a**) Chroma C, (**b**) total color difference. LS—*Latilactobacillus sakei* ATCC 15521; LP—*Lactipalntibacillus plantarum* ATCC 4080; LB—*Levilactobacillus brevis* DSM 20053; LR—*Limosilactobacillus reuteri* DSM 17938; LF—*Fructilactobacillus fructivorans* DSM 20203; a–p—results marked with the same letters are not statistically significantly different at α = 0.05.

Saturation, also called chroma values (C*), indicates the intensity of the color of the sample [31,35,38]. Following thermal treatment in pumpkin juices, an increase in saturation was observed. The behavior varied depending on the type of juice after fermentation; non-thermally-treated juices remained more stable, with no change or increase in saturation, regardless of the LAB strain used. Analysis of heat-treated juices revealed a decrease in saturation, with a greater decrease observed with longer fermentation processes (Figure 2a). Furthermore, when calculating the total color difference values, significant changes were observed compared to fresh pumpkin juice (Figure 2b).

In Figure 2b, the red line denotes the maximum value beyond which a non-expert person would perceive a difference with the naked eye. Our findings demonstrate that, during fermentation, consumers could visually identify all juices, despite changes in color coordinates (L, a*, and b*), as being identical to raw pumpkin juice. The only significant contrast was observed early in the process between raw (J_D0) and raw heat-treated juice (J_P_D0).

That observation of dE typically varies based on the specific conditions of the process and the strains of bacteria involved. Sun et al. [6] conducted experiments with various lactobacillus strains (*Lacticaseibacillus casei* ATCC334 (Lc), *Lactiplantibacillus plantarum* CICC20265 (Lp), *Lactobacillus acidophilus* CGMCC1.2686 (La), *Lacticaseibacillus paracasei* CICC20245 (Lpc), and *Lactobacillus helveticus* CICC6024 (Lh)) in the fermentation of pumpkin juice over 48 h. They observed significant differences (ranging from 40 to 80) between raw and fermented pumpkin juice. The smallest difference was noted for La and Lh strains (dE = 40), and the highest was observed for Lp. Our study found the lowest differences after seven days of fermentation for *Limosilactobacillus reuteri* DSM 17938 and LF—*Fructilactobacillus fructivorans* DSM 20203 strains.

### 2.3. Sugar Content

**Figure 3:**
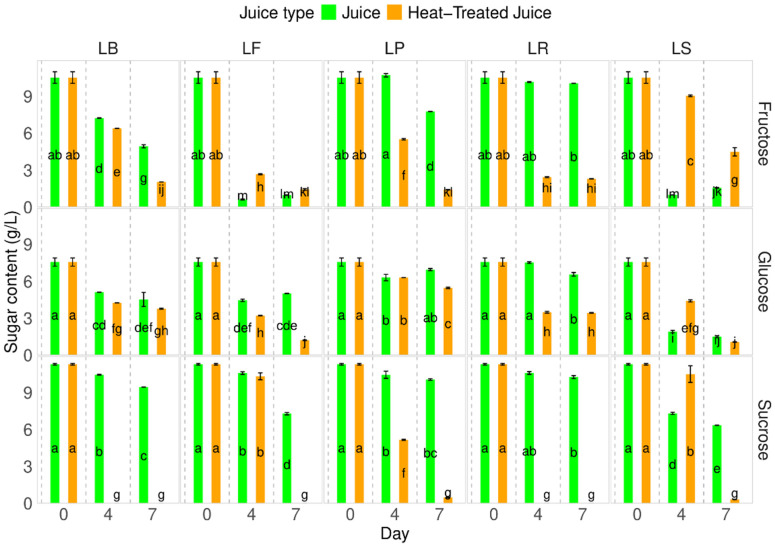
Influence of heat-treatment and fermentation on the sugar content. LS—*Latilactobacillus sakei* ATCC 15521; LP—*Lactipalntibacillus plantarum* ATCC 4080; LB—*Levilactobacillus brevis* DSM 20053; LR—*Limosilactobacillus reuteri* DSM 17938; LF—*Fructilactobacillus fructivorans* DSM 20203; a–m—results marked with the same letters are not statistically significantly different at α = 0.05.

Identifying and quantifying sugars in heat-treated pumpkin juices showed no changes in sugar types and content (Figure 3). The fermentation process caused a decrease in sugar content. The decrease was influenced by the juice type; a lower decrease in sucrose content but higher in fructose and glucose were observed in the juice after heat-treatment. Each lactobacillus strain used behaved in different ways. The highest decrease in fructose was observed for LF—*Fructilactobacillus fructivorans* DSM 20203, while for that species, fructose is the most valuable source of carbon.

In our research, different percentages of sucrose/glucose/fructose were observed compared to raw pumpkin juices presented by Zhang et al. [35]. In their study of *Cucurbita moschata*, they found sugar contents of 2.14 g/100 g f.w. for sucrose, 1.08 g/100 g f.w. for glucose, and 1.72 g/100 g f.w. for fructose.

It has been noted that the reduction in sugar content is consistent with the findings of Sun et al. [6], who observed a decrease in total sugar content from 42 to 20–25 g/L. They explained that sugars are the primary carbon source for lactic acid bacteria during fermentation. Other researchers have reported similar observations regarding other types of juices [40,41].

### 2.4. Antioxidant Properties of Pumpkin Juices

The antioxidant properties of a substance can be assessed using various methods, most of which involve the reduction of free radicals by antioxidant compounds. While the FRAP method is widely accepted for evaluating the activity of an antioxidant by its ability to reduce Fe(III), it may not encompass all antioxidants capable of inactivating free radicals. Similarly, the TEAC value, which quantifies an antioxidant’s ability to scavenge the cation radical ABTS+, is determined by measuring the reduction in absorbance due to the influence of the antioxidant relative to the activity of the reference antioxidant [42]. That is the reason for using few methods to describe products’ antioxidant properties.

Regardless of pre-treatment, fermentation day, and strain used, the antioxidant capacity of pumpkin juices to reduce ferricyanide did not differ, and the antioxidant potential ranged from 43 to 69 mg TE/g. dm (Table 3). The ABTS and polyphenol content methods indicated increased antioxidant power during fermentation. Among non-heat-treated juices, strains LP and PR exhibited the highest values in both methods, consistent with the findings Sun et al. [6]. Their research suggested that *L. plantarum* had the highest antioxidant capacities after fermenting pumpkin juices with different strains, potentially linked to vanillic acid and sinapic acid content.

Kulczyński et al. [42] observed similar values for ABTS for *C*. *moschiata* in fresh pumpkin extracts. Sun et al. [6] noted an increase in polyphenol and antioxidant properties (measured via DPPH) during fermentation but a decrease in measurement in another method, ABTS*. Conversely, Wang et al. [43] reported no change in total polyphenol content before and after pumpkin juice fermentation, but they did observe a twofold increase in flavonoid content.

### 2.5. Carotenoid Content in Pumpkin Juices

**Figure 4:**
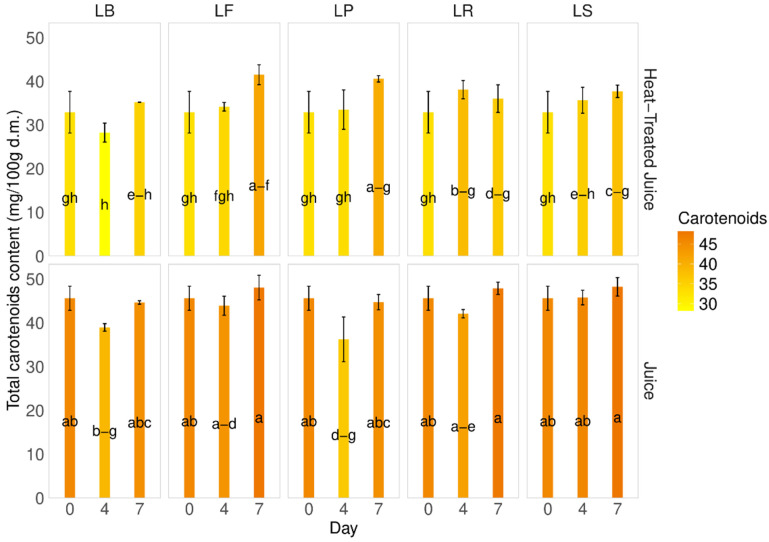
Total carotenoid content. LS—Latilactobacillus sakei ATCC 15521; LP—*Lactipalntibacillus plantarum* ATCC 4080; LB—*Levilactobacillus brevis* DSM 20053; LR—*Limosilactobacillus reuteri* DSM 17938; LF—*Fructilactobacillus fructivorans* DSM 20203; a–h—results marked with the same letters are not statistically significantly different at α = 0.05.

Carotenoids, the main pigment present in pumpkins and their juice, were found to be 50.3 ± 8.64 mg of β-carotene/100 g d.m. in the pumpkin samples and 45.6 ± 2.8 mg of β-carotene/100 g d.m. in the fresh juice. After heat treatment of the juice, a decrease in carotenoid content was observed (32.9 4.8 mg of β-carotene/100 g d.m.) (Figure 4). A slight decrease in carotenoid content was noted during fermentation on the 4th day. Interestingly, the total carotenoid content during fermentation in juices without heat treatment was higher than in heat-treated juices, with a difference of approximately 18% and a 7.5–28% decrease. The smallest changes were observed for strain LP on the 4th and 7th days (7.5–9.4%). Statistical analysis showed no significant changes during the fermentation process for each tested juice, which is promising, considering carotenoids in pumpkins are the main sources of active substances.

The carotenoid content in pumpkins can vary among different cultivars [6,35,44]. Factors such as production methods, cultivation, storage, and thermal treatment can also influence carotenoid levels. Zdunić et al. [45] observed a decrease in carotene content from raw pumpkin (86.3 µg/g) to wine (27.6 µg/g), jam (63.9 µg/g), and juice (28.6 µg/g) due to a negative influence of thermal treatment. A similar trend was observed during the fermentation process. Wang et al. [43], observed a decrease in β-carotene content in pumpkin juices from 3.11 to 2.48 mg/L during fermentation. Similarly, our previous studies on carrot juice fermentation showed a significant decrease in carotenoid content during the first day of the process [37]. This contrasts the presented research on pumpkin juices with different LAB strains. Here, thermal treatment affects the carotenoid content, since heat negatively affects carotene [39]. A similar trend is observed during fermentation, although the decrease is less significant than during heating.

## 3. Materials and Methods

### 3.1. Material

The test material consisted of various Muscat pumpkins (*Cucurbita moschata*) purchased at a local market in Warsaw. The pumpkins were stored at 2–8 °C for a maximum of 3 days before use. A total of 3 kg of peeled and cut pumpkins were used for juice preparation. The biological material included five strains of bacteria: *Latilactobacillus sakei* ATCC 15521 (LS), *Lactiplantibacillus plantarum* ATCC 4080 (LP), *Levilactobacillus brevis* DSM 20053 (LB), *Limosilactobacillus reuteri* DSM 17938 (LR), and *Fructilactobacillus fructivorans* DSM 20203 (LF). These strains were obtained from the American Type Culture Collection (ATCC, Manassas, VA, USA) and the German Collection of Microorganisms and Cell Cultures GmbH (DSMZ, Braunschweig, Germany).

### 3.2. Technological Processing

#### 3.2.1. Juice Preparation

Before processing, the pumpkins were washed in water at room temperature. They were peeled with a knife and cut into small pieces. The juice was extracted by pressing the raw vegetable pieces using a commercial single-screw juicer (NS-621CES; Kuvings, Daegu, Republic of Korea), which separated the juice from the pomace. The obtained juice was bottled under sterile conditions in 50 mL bottles.

#### 3.2.2. Juice Heat Treatment

According to the methodology presented by Zhang et al. [46] and Adiara et al. [47], and based on our previous tests (unpublished data), we used heat treatment with the selected time and temperature. Those parameters guaranteed a decrease in bacteria and mold count. Half the juice bottles were heat treated at 80 °C for 40 min in an autoclave (HG-80, HMC Europe GmbH, Tüssling, Germany) to eliminate native microbiota. After heat treatment, the juice was cooled down to 20 °C.

#### 3.2.3. Fermentation Process

Prior to testing, the strains were stored in a frozen state. The bacteria were then cultivated on MRS medium and subsequently subjected to centrifugation and two washes with saline solution. Next, the inoculum was prepared in 0.85% NaCl. The lactic acid bacteria (LAB) was prepared, and the optical density was adjusted to 0.5 ° McF on the McFarland scale (Densimat, BioMerieux, Craponne, France), corresponding to approximately 1 × 10^8^ (CFU/mL), and was used for fermentation. To promote salt-tolerant LAB bacteria growth and inhibit pathogenic microflora growth, 2% *m*/*v* NaCl was added directly to the cooled juice before the inoculum addition. Then, each pumpkin juice was inoculated with a 1% (*v*/*v*) solution of the strain. Fifty mL bottles were closed and placed in an incubator (BD-S115, Binder, Tuttlingen, Germany). A separate sample was prepared for each day of the experiment and each strain. The fermentation process was carried out for seven days at 25 °C. The tests were conducted on the process’s 0, 4th, and 7th days.

### 3.3. Analytical Methods

#### 3.3.1. Determination of the Count of Lactic Acid Bacteria

The total count of LAB was calculated using the pour plate method to enumerate viable cells. Juice samples were serially diluted using sterile saline (0.85% NaCl, Biomaxima, Lublin, Poland). The samples were streaked onto de Man Rogosa and Sharpe Agar (MRS, Biomaxima, Lublin, Poland) plates and incubated at 28 °C ± 2 °C for 48 h ± 4 h. The number of colonies grown was counted (ProtoCOL 3—automatic colony counting and zone measuring, Synbiosis, Frederick, MD, USA) and recorded as log CFU/mL. The analysis was performed after inoculation (for samples with the addition of lactic acid bacteria) and on each selected day of fermentation.

#### 3.3.2. Determination of the Total Viable Count

Unpasteurized and pasteurized juice was tested for the total viable count (TVC) of microorganisms. TVC was enumerated on plate count agar (PCA, Biomaxima, Lublin, Poland) and incubated at 30 °C for 72 h. The TVC was counted using a ProtoCOL 3 (Synbiosis, Cambridge, UK) and determined in log CFU/mL.

#### 3.3.3. Dry Matter

The dry matter was determined by the gravimetric method. Approximately 0.6–1 g of sample was placed in a dish with filter paper and dried using a vacuum drying method (Memmert VO400, Schwabach, Germany) under a pressure of 10 mPa at 75 °C for 24 h until a constant weight was achieved.

#### 3.3.4. Solid Soluble Content

The solid soluble content was determined by refractometer using a small pocket refractometer PAL-3 (ATAGO Instruments, Tokyo, Japan) at a temperature of 25 °C.

#### 3.3.5. pH and Total Acidity

The pH value of the juice was measured using a pH meter (S-210, Mettler Toledo, Greifensee, Switzerland); the temperature for the test was 25 °C.

Total acidity was determined by potentiometric titration with a 0.1 M NaOH solution until pH 8.1 was reached. The weighed samples were diluted with distilled water to a volume of 50 L. Then, the sodium hydroxide was added to the 25 mL of the samples until the pH reached 8.1. The total acidity and lactic acid content per 100 g of product were determined based on the results and calculated with the formula presented by Wierzbicka and Janiszewska-Turak [47].

(1) Total acidity=V1·0.1·0.9·V2·100/25·mg lactic acid100g product

where *V*_1_—the volume of NaOH from the burette (mL); *V*_2_—the total volume of the filtrate (mL); *m*—a mass of sample (g); 0.9—conversion of total acidity to acidity in relation to lactic acid; 0.1—molality of NaOH; 25—sample volume (mL).

#### 3.3.6. Color

The color coordinates of the juice were determined using a CR-5 spectrophotometer (Konica Minolta Sensing Inc., Osaka, Japan) in the CIE L*a*b* system, with the following parameters: illumination D65, angle 2°. The equipment was calibrated with black and white standards according to the methodology described by Janiszewska-Turak et al. [37]. All measurements were performed in ten repetitions. Also, the total color difference (dE) and chroma (C*) were calculated from equations presented by Zhang et al. [35]. The raw pumpkin juice was used as a reference value of L, a*, and b* for the total color difference.

#### 3.3.7. HPLC Analysis of Sugars

Sugar concentration was performed according to the EN 12630:1999 standard methodology [48] using a refractive index detector (Waters 2414, Milford, CT, USA). The analysis was performed isocratically with 0.1 mM calcium disodium EDTA. The analysis of sugars was carried out on a Sugar-Pak I, 10 μm, 6.5 mm × 300 mm analytical column with a Sugar-Pak and Guard-Pak insert, 10 μm (both Waters, Milford, USA) at a column temperature of 90 °C and a detector temperature of 35 °C. The samples were analyzed within 20 min at a 0.5 mL/min flow rate.

#### 3.3.8. Total Phenolic Content

The total phenolic content in pumpkin juices was determined using a spectrophotometric method involving a color reaction with Folin-Ciocalteu reagent [49]. The extracts were diluted with 80% ethanol solution, and the reactions were conducted in 96-well plates. First, 40 µL of a 5-fold diluted Folin-Ciocalteu reagent was added to 10 µL of the extract. After 3 min, 250 µL of a 7% sodium carbonate solution was added. The solution was incubated for 60 min at 25 °C in the dark. Absorbance was measured at 750 nm using a Multiskan Sky plate reader (Thermo Electron Co., Waltham, MA, USA). The absorbance of a blank sample, where the extraction reagent replaced the extract, was also measured. Each extract was tested in duplicate. A chlorogenic acid (Sigma Aldrich, Buchs, Switzerland) calibration curve in the 0–100 µg/mL concentration range was prepared to quantify the polyphenol content. Results were expressed as milligrams of chlorogenic acid per 100 g of dry matter.

#### 3.3.9. Antioxidant Properties

Spectrophotometric methods were used to assess the antioxidant properties of the samples, which consisted of determining the ability to reduce Fe^3+^ ions (RP) and the cationic radical 2,2-azynobis(3-ethylbenzothiazoline-6-sulfonate) (ABTS+).

For antioxidant activity assessment, the ABTS radical solution was prepared by dissolving ABTS (abcr GmbH, Karlsruhe, Germany) and potassium persulfate (Sigma Aldrich, Saint Louis, MO, USA) in distilled water and storing it at 4 °C for 16 h. Before analysis, the ABTS radical solution was diluted with 80% ethyl alcohol (Chempur, Piekary Śląskie, Poland) to achieve an absorbance value between 0.680 and 0.720, measured at 734 nm. Sample extracts (10 µL) were mixed with 250 µL of the radical working solution in 96-well plates and incubated for 6 min at 25 °C [50]. The absorbance of the reaction mixture was measured using a Multiskan Sky plate reader (Thermo Electron Co., St. Louis, MO, USA) against a blank (without the sample extract). Antioxidant activity (the degree of radical scavenging in the presence of the antioxidant) was measured in duplicate for each sample. Results were expressed as mg of Trolox per 1 g of dry matter.

##### Iron Ion Reduction Method

To determine the reducing power (RP) of iron ions by the analyte, 25 μL of the extract, 50 μL of a 1% aqueous solution of potassium ferricyanide, and 75 μL of distilled water were pipetted into a well. The mixture was thoroughly mixed and then incubated at 50 °C for 20 min (INCU-Line ILS 10; VWR, Radnor, PA, USA). After incubation, 50 μL of 10% trichloroacetic acid was added. Subsequently, 100 μL of the reaction mixture was transferred to a new well, followed by adding 100 μL of distilled water and 20 μL of a 0.1% iron (III) chloride solution, and the mixture was remixed. After 10 min, absorbance was measured at 700 nm against a blank. The RP value was expressed as mg of Trolox [51].

#### 3.3.10. Carotenoids Content

The total carotenoid content (TCC) was measured according to the methodologies described in references [52,53] based on spectrophotometric measurements. A specific wavelength of 450 nm was used for carotenoid detection (Spectronic 200; Thermo Fisher Scientific Inc., Waltham, MA, USA). The pumpkin juice was extracted twice with acetone and petroleum ether. The blank absorbance for the ether was measured at 450 nm. The results were shown as mg β-carotene/100 g of juice dry substance.

### 3.4. Statistical Treatment

The results obtained were subjected to statistical analysis using Statistica 13 software (StatSoft, Warsaw, Poland). An analysis of variance (ANOVA) was performed to assess the significance of the physical and chemical properties of the pumpkin. The Tukey HSD method divided results into homogeneous groups (α = 0.05). The other parameters were determined using MS Excel 2019 and R program (plots).

## 4. Conclusions

The main objective of this study was to assess the impact of a particular *Lactobacillus* strain on the lactic fermentation process of pumpkin juice. The findings indicate that both the *Latilactobacillus sakei* and *L. plantarum* strains were effective in fermenting pumpkin juice. *L. sakei* exhibited higher adaptability, resulting in higher bacterial counts after fermentation. This finding partially supports the initial hypothesis, suggesting that although both strains yielded comparable fermentation outcomes, *L. sakei* exhibited a distinctive capacity for bacterial growth.

The study confirmed the expected decrease in pH and increase in total acidity, which is characteristic of lactic acid fermentation in vegetable juices. Furthermore, as demonstrated by the extract and sugar analysis, the reduction in sugar content validated the anticipated metabolic activity during fermentation. The investigation into the type of sugar present in the raw material also provided insight into the fermentation dynamics.

Furthermore, the study investigated consumer-relevant attributes, including color, antioxidant capacity, and total carotenoids. While the heat treatment process reduced carotenoid content, the fermentation process, particularly with *L. plantarum*, was observed to enhance the antioxidant potential of the juice. Despite observable color changes (dE values below 5), consumers could not distinguish between raw and fermented pumpkin juices, indicating that fermentation did not significantly alter the product’s visual appeal.

The second objective was to evaluate the influence of heat treatment on the physical and chemical characteristics of fermented pumpkin juice. The findings indicate that heat treatment did not substantially influence the key parameters, including dry matter and extract content. This finding supports the second hypothesis, indicating that heat treatment does not significantly alter the physical and chemical characteristics of the juice during fermentation. However, it was observed that heat treatment reduced carotenoid levels, indicating a trade-off between enzyme deactivation and nutrient retention.

In conclusion, the findings of this study confirm that the selected *Lactobacillus* strains are effective for the lactic fermentation of pumpkin juice, with *L. sakei* exhibiting a notable advantage in bacterial proliferation. While heat treatment did not significantly impact the essential physical and chemical attributes of the juice, it did influence the carotenoid content, which could be a factor to consider in product formulation, depending on the desired nutritional profile. The extended fermentation period observed indicates that further optimization may be necessary to enhance efficiency.

## Figures and Tables

**Table 1:** Influence of thermal treatment and strain type during fermentation on the selected physical and chemical parameters of pumpkin juice.

Juice Type	Strain	Day	Symbol	Extract (°Brix)	Dry Matter (%)	pH (-)	Total Acidity (g Lactic Acid/100 g Juice)
J		0	J__D0	6.75 ± 0.05 a	6.31 ± 0.09 a	6.23 ± 0.02 a	3.57 ± 0.21 f–i
	LB	4	J_LB_D4	6.60 ± 0.04 b	5.61 ± 0.03 b–e	3.73 ± 0.01 d–h	3.81 ± 0.13 f–i
		7	J_LB_D7	5.10 ± 0.01 k	5.41 ± 0.73 de	3.65 ± 0.02 f–j	7.08 ± 1.03 bc
	LF	4	J_LF_D4	6.00 ± 0.04 def	5.16 ± 0.07 ef	3.70 ± 0.04 e–i	4.91 ± 0.02 e–f
		7	J_LF_D7	4.85 ± 0.05 l	4.73 ± 0.07 fg	3.58 ± 0.01 ij	7.73 ± 0.41 bc
	LP	4	J_LP_D4	5.90 ± 0.02 fg	5.44 ± 0.09 de	3.46 ± 0.05 kl	5.36 ± 0.04 de
		7	J_LP_D7	4.80 ± 0.03 l	4.28 ± 0.06 g	3.41 ± 0.01 l	7.93 ± 0.82 b
	LR	4	J_LR_D4	5.50 ± 0.02 i	4.82 ± 0.06 fg	3.68 ± 0.01 f–i	3.58 ± 0.39 f–i
		7	J_LR_D7	5.20 ± 0.03 j	4.28 ± 0.21 g	3.65 ± 0.01 f–i	6.56 ± 0.11 c
	LS	4	J_LS_D4	5.90 ± 0.01 g	5.63 ± 0.01 b–e	3.58 ± 0.04 ki	5.11 ± 0.04 de
		7	J_LS_D7	5.95 ± 0.05 efg	5.52 ± 0.08 cde	3.55 ± 0.02 jk	9.15 ± 0.35 a
HT_J		0	HT_J_D0	6.65 ± 0.05 ab	6.29 ± 0.13 a	6.21 ± 0.08 a	1.65 ± 0.28 j
	LB	4	HT_J_LB_D4	6.10 ± 0.03 cd	6.17 ± 0.18 ab	3.79 ± 0.04 cde	3.08 ± 0.35 i
		7	HT_J_LB_D7	6.00 ± 0.04 cde	6.08 ± 0.13 abc	3.74 ± 0.04 def	4.38 ± 0.15 e–h
	LF	4	HT_J_LF_D4	6.15 ± 0.05 c	5.90 ± 0.01 a–d	3.86 ± 0.01 bc	3.46 ± 0.48 ghi
		7	HT_J_LF_D7	5.70 ± 0.02 h	5.45 ± 0.03 acde	3.63 ± 0.03 g–j	5.08 ± 0.20 de
	LP	4	HT_J_LP_D4	5.95 ± 0.05 efg	5.82 ± 0.06 a–d	3.60 ± 0.01 hij	4.46 ± 0.19 e–g
		7	HT_J_LP_D7	6.10 ± 0.05 cd	5.96 ± 0.09 a–d	3.56 ± 0.04 jk	6.15 ± 0.27 cd
	LR	4	HT_J_LR_D4	6.10 ± 0.01 cd	6.07 ± 0.01 abc	3.92 ± 0.02 b	3.23 ± 0.08 hi
		7	HT_J_LR_D7	6.10 ± 0.03 cd	6.03 ± 0.02 abc	3.80 ± 0.04 cde	4.92 ± 0.07 ef
	LS	4	HT_J_LS_D4	5.95 ± 0.05 efg	5.78 ± 0.08 a–d	3.82 ± 0.02 bcd	4.70 ± 0.02 e–f
		7	HT_J_LS_D7	5.90 ± 0.03 efg	5.73 ± 0.02 a-d	3.73 ± 0.03 def	6.12 ± 0.38 cd

J—juice; HT—heat-treatment; D—day; LS—*Latilactobacillus sakei* ATCC 15521; LP—*Lactipalntibacillus plantarum* ATCC 4080; LB—*Levilactobacillus brevis* DSM 20053; LR—*Limosilactobacillus reuteri* DSM 17938; LF—*Fructilactobacillus fructivorans* DSM 20203. a–l—results marked in columns with the same letters are not statistically significantly different at α = 0.05.

**Table 2:** Color coefficients of fermented pumpkin juices.

Juice Type	Strain	Day	Symbol	L* Lightness	a* Redness	b* Yellowness
J		0	J_D0	32.84 0.07 bc	11.17 0.02 jk	27.94 0.24 jkl
	LB	4	J_LB_D4	31.94 0.05 hi	11.52 0.05 g–j	28.54 0.24 hij
		7	J_LB_D7	32.46 0.27 de	10.60 0.10 l	26.17 0.57 n
	LF	4	J_LF_D4	32.36 0.14 ef	10.96 0.0 kl	28.41 0.12 h–k
		7	J_LF_D7	32.50 0.18 de	11.11 0.26 jk	27.68 0.33 kl
	LP	4	J_LP_D4	32.06 0.11 ghi	11.86 0.10 fgh	29.16 0.28 fgh
		7	J_LP_D7	31.30 0.18 k	11.79 0.27 f–i	29.62 0.76 fg
	LR	4	J_LR_D4	32.24 0.18 efg	11.32 0.07 jk	28.84 0.23 ghi
		7	J_LR_D7	32.94 0.29 b	11.37 0.16 ijk	27.19 0.48 lm
	LS	4	J_LS_D4	32.30 0.17 efg	11.88 0.11 fg	29.81 0.28 ef
		7	J_LS_D7	31.96 0.35 hi	12.16 0.15 def	29.57 0.59 fg
HT_J		0	HT_J_D0	34.55 0.36 a	15.57 0.40 a	33.10 0.53 a
	LB	4	HT_J_LB_D4	31.87 0.07 i	13.40 0.10 b	32.34 0.18 ab
		7	HT_J_LB_D7	32.66 0.04 cd	11.28 0.15 jk	28.15 0.37 ijk
	LF	4	HT_J_LF_D4	32.15 0.08 fgh	12.65 0.17 c	31.35 0.32 cd
		7	HT_J_LF_D7	32.14 0.14 fgh	11.45 0.14 hij	28.77 0.07 g–j
	LP	4	HT_J_LP_D4	31.60 0.05 j	12.47 0.12 cde	32.16 0.31 bc
		7	HT_J_LP_D7	32.49 0.15 de	11.19 0.18 jk	29.19 0.41 fgh
	LR	4	HT_J_LR_D4	33.03 0.05 b	12.55 0.12 cd	31.09 0.09 d
		7	HT_J_LR_D7	32.34 0.15 ef	10.67 0.24 l	26.79 0.14 mn
	LS	4	HT_J_LS_D4	32.93 0.31 b	12.12 0.28 ef	28.58 0.18 hij
		7	HT_J_LS_D7	31.20 0.04 k	11.40 0.25 ij	30.64 0.31 de

J—juice; HT—heat-treatment; D—day; LS—*Latilactobacillus sakei* ATCC 15521; LP—*Lactipalntibacillus plantarum* ATCC 4080; LB—*Levilactobacillus brevis* DSM 20053; LR—*Limosilactobacillus reuteri* DSM 17938; LF—*Fructilactobacillus fructivorans* DSM 20203. a–n—results marked in columns with the same letters are not statistically significantly different at α = 0.05.

**Table 3:** Influence of thermal treatment and strain type during fermentation on pumpkin juice’s polyphenol content and antioxidant properties.

Juice Type	Strain	Day	Symbol	Reducing Power (mg TE/g d.m.)	ABTS (mg TE/g Juice d.m.)	Polyphenols (mg Chlorogenic Acid/100 g Juice d.m.)
J		0	J__D0	43.97 ± 3.86 a	1.10 ± 0.09 d	75.08 ± 7.38 ab
	LB	4	J_LB_D4	44.85 ± 0.90 a	1.65 ± 0.25 cd	100.61 ± 5.92 ab
		7	J_LB_D7	57.37 ± 5.08 a	14.56 ± 0.05 a	169.26 ± 7.41 ab
	LF	4	J_LF_D4	54.95 ± 2.17 a	2.05 ± 0.17 cd	97.62 ± 3.00 ab
		7	J_LF_D7	53.67 ± 5.56 a	16.57 ± 0.12 a	186.00 ± 7.67 a
	LP	4	J_LP_D4	49.26 ± 1.98 a	1.34 ± 0.10 cd	120.87 ± 4.77 ab
		7	J_LP_D7	69.44 ± 5.33 a	18.27 ± 0.14 a	210.86 ± 7.18 a
	LR	4	J_LR_D4	50.80 ± 4.72 a	1.19 ± 0.07 cd	114.67 ± 13.26 ab
		7	J_LR_D7	55.28 ± 2.87 a	18.20 ± 0.05 a	208.00 ± 6.96 a
	LS	4	J_LS_D4	44.63 ± 2.78 a	1.31 ± 0.12 cd	84.49 ± 5.67 ab
		7	J_LS_D7	56.12 ± 3.69 a	12.66 ± 2.18 ab	162.9111.47 ab
HT_J		0	HT_J_D0	42.78 ± 2.87 a	0.95 ± 0.01 d	67.63 ± 1.38 ab
	LB	4	HT_J_LB_D4	52.62 ± 4.09 a	1.15 ± 0.08 cd	71.38 ± 6.76 ab
		7	HT_J_LB_D7	53.94 ± 2.57 a	10.69 ± 0.59 ab	135.76 ± 1.59 ab
	LF	4	HT_J_LF_D4	47.24 ± 3.58 a	1.30 ± 0.04 cd	73.48 ± 2.85 ab
		7	HT_J_LF_D7	48.57 ± 1.76 a	12.42 ± 0.43 ab	99.95 ± 5.37 ab
	LP	4	HT_J_LP_D4	57.75 ± 3.72 a	1.34 ± 0.27 cd	102.16 ± 8.97 ab
		7	HT_J_LP_D7	52.91 ± 0.46 a	10.70 ± 0.87 ab	176.92 ± 3.57 ab
	LR	4	HT_J_LR_D4	47.38 ± 0.96 a	1.21 ± 0.16 cd	79.50 ± 3.52 ab
		7	HT_J_LR_D7	53.79 ± 0.50 a	10.27 ± 0.95 ab	138.13 ± 5.87 ab
	LS	4	HT_J_LS_D4	47.46 ± 3.07 a	1.04 ± 0.16 d	95.44 ± 4.99 ab
		7	HT_J_LS_D7	44.93 ± 2.80 a	12.59 ± 1.29 bc	131.50 ± 8.20 ab

J—juice; HT—heat-treatment; D—day; LS—*Latilactobacillus sakei* ATCC 15521; LP—*Lactipalntibacillus plantarum* ATCC 4080; LB—*Levilactobacillus brevis* DSM 20053; LR—*Limosilactobacillus reuteri* DSM 17938; LF—*Fructilactobacillus fructivorans* DSM 20203. a–d—results marked in columns with the same letters are not statistically significantly different at α = 0.05.

## Data Availability

Data available for request from the corresponding author.
